# The Stress–Strain State in the Pelvis During Sit-to-Stand Transfer

**DOI:** 10.3390/bioengineering12121328

**Published:** 2025-12-05

**Authors:** Urban Žnidaršič, Andrej Žerovnik, Matevž Tomaževič, Robert Kunc

**Affiliations:** 1 Chair of Modeling in Engineering Sciences and Medicine, Faculty of Mechanical Engineering, University of Ljubljana, Kongresni trg 12, 1000 Ljubljana, Slovenia; 2 Division of Surgery, Department of Traumatology, University Medical Centre Ljubljana, Zaloška cesta 7, 1000 Ljubljana, Slovenia

**Keywords:** pelvis, structural analysis, sit-to-stand transfer, finite element method, inverse dynamics, muscle forces

## Abstract

To achieve early mobilization of patients with unstable pelvic fractures, the osteosynthesis methods used must withstand the loads in the pelvis during everyday movements. There is currently no predictive tool to assess how suitable these methods are for this purpose. The development of such a tool requires an understanding of the effects of joint and muscle loads on the structural behavior of the pelvis during movement. To further this cause, the stress–strain state of the pelvis during a sit-to-stand transfer of a healthy adult male was analyzed. Muscle and joint reaction forces during the motion were predicted using a rigid-body musculoskeletal model. These loads were then utilized in the first-ever dynamic structural analysis of the pelvis during a sit-to-stand transfer using the finite element method. Several similarities in stress distributions during sit-to-stand transfer, gait, and standing were identified by comparing the finite element analysis results with literature. The common areas of increased stress between the three motions are the acetabular notch, the superior edge of the obturator foramen, the attachments of the gluteus maximus on the ilium, and the lesser sciatic notch. The results also provide important insights into global behavior of the pelvic ring and indicate the locations of concentrated stress during sit-to-stand transfer.

## 1. Introduction

Pelvic ring fractures occur in 17–35/100,000 individuals/year. While unstable pelvic ring disruptions are relatively rare, their treatment is a complex and still-developing area of modern traumatology. It can take up to 12 weeks before patients can independently bear their full weight, and upwards of 30% of patients suffer from persistent and significant chronic pain and/or urogenital problems [1].

The predominant method for treatment of unstable pelvic fractures and dislocations is a surgical procedure called osteosynthesis, where injured bones are stabilized with various internal fixation approaches and materials [2,3,4]. However, the optimal procedures for specific injuries have not yet been agreed upon [4,5,6,7]. Trauma surgeons must therefore rely on experience instead of biomechanically validated methods. Tested and proven guidelines or tools for selecting fixation methods for specific pelvic fractures would expedite patient mobilization and reduce rehabilitation times.

To facilitate early mobilization, stabilized injuries and osteosynthesis materials should provide sufficient support to withstand loads from various body movements (walking, ascending and descending stairs, sit-to-stand transfer, etc.) that the patient will perform. Evaluating different fixation methods will therefore require an assessment of loads in the human body and a method for predicting the behavior of stabilized injuries under these loads.

Measuring joint loads in vivo is possible, but the process is complex and only situationally appropriate. They have been measured in patients after hip [8,9] and vertebrae replacement surgeries [10,11] using instrumented implants with telemetric data transmission. Measuring muscle forces in vivo, however, has been even rarer, as ethical concerns and the need to ensure the comfort of research volunteers currently prevent us from performing such experiments. The closest alternative to measuring muscle forces is measuring muscle activation with methods such as electromyography (EMG) and muscle contraction (MC) sensors [12] and using the data to directly calculate muscle forces [13,14,15] or predict them with deep learning methods [16,17]. As an alternative to in vivo measurements, computational methods in this field have been increasing in use. Rigid-body musculoskeletal model analyses using inverse dynamics methods have become the standard for predicting muscle and joint loads during multi-joint movements, as they are more flexible and simpler to use than the experimental methods mentioned above. These analyses are enabled by dedicated software such as OpenSim [18,19], which also provides databases containing several open-source models and datasets.

Once muscle and joint loads are determined, their effect on the human body must be assessed. Using cadavers is a well-established technique [20,21] that presents challenges regarding cost, sample availability, safety, and reproducibility of results. Artificial bone substitutes are being developed as an alternative [22]. However, applying time-dependent muscle forces remains complex when performing physical experiments. Alternatively, computational analyses using the finite element method (FEM) enable detailed geometry and material model definition, which can be based on medical imaging data of individuals [23]. The FEM also offers a relatively straightforward way to change boundary conditions. This method has seen heavy use in biomechanics research and has been developed to the point where complex and comprehensive human body models with non-linear material characteristics have been created [24].

Connecting muscle and joint loads estimated with inverse dynamics methods to FE models for structural analysis of the pelvis has, to our knowledge, only been attempted a handful of times [25,26,27]. These studies examined gait, and only one used time-dependent loading conditions [26], while others used static analysis of only certain gait stages [25,27]. Each study utilized a separate musculoskeletal gait model with a different set of muscles included. The FE models differed as well: some described the pelvic bone as a homogenous material [26,27], while Watson et al. [25] considered the differences between the cortical and trabecular bone, and also included ligaments.

The purpose of this study was to provide, to our knowledge, the first dynamic finite element analysis of the structural response of the pelvis during the sit-to-stand (STS) transfer using physiologically informed muscle and joint forces. By demonstrating the behavior of the pelvis during this everyday motion, we address an important gap in current biomechanical knowledge and take a step towards the development of medical tools for predictive modeling of subject-specific treatment of unstable pelvic ring fractures.

## 2. Materials and Methods

Firstly, a suitable musculoskeletal model and kinematics data of a sit-to-stand transfer had to be obtained. Next, muscle forces and hip and spine reaction forces during movement were estimated in OpenSim v4.4. For the FEM analysis, the pelvic bones and ligaments were extracted and modified from the average adult male (AM50) pedestrian in v4.02 of the Total HUman Model for Safety (THUMS) data set. Finally, boundary conditions based on results from OpenSim were applied to the FE model, and a dynamic structural FEM analysis was performed with LS-DYNA R12.

### 2.1. Motion Analysis

#### 2.1.1. Sit-to-Stand Transfer Data and Musculoskeletal Model

**Figure 1:**
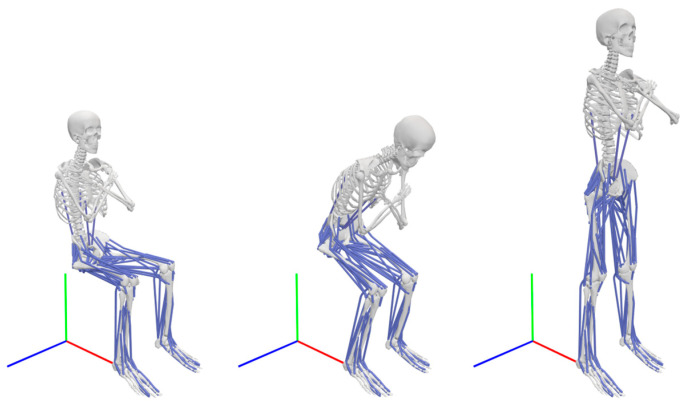
The scaled Full Body Model 2016 in OpenSim during the STS transfer. The arms are crossed, with hands resting on the shoulders. The global coordinate system is shown at the feet of the model, with the *x*-axis colored red, the *y*-axis colored green, and the *z*-axis colored blue.

The model used for the STS transfer movement analysis was the Full Body Model 2016, developed by Caruthers et al. [28] specifically for that purpose. The model includes 46 degrees of freedom (DOF) and 94 muscles, represented by 194 Hill-type muscle-tendon actuators. Sixty of these actuators, representing 42 muscles on both the left and right sides of the body, attach to the pelvis. The model has certain simplifications regarding the muscles and their attachments. The rectus abdominis, for instance, terminates before reaching the pelvis, and only two actuators represent the complex musculature of the back. The list of muscles attached to one side of the pelvis with a corresponding number of actuators is presented in Table 1. The Full Body Model 2016 is pictured in Figure 1.

Caruthers et al. [28] measured the sit-to-stand transfer of seven healthy, young adults. A scaled version of the base model was created for each participating individual. The degrees of freedom for the scaled models were reduced by locking the arm coordinates, decreasing the total DOF count to 19. The base, unscaled model, motion data (marker trajectories and ground reaction forces), and the scaled models for four individuals were made freely available under the Creative Commons license. The data set chosen for research belonged to a healthy young male participant with a height of 181 cm and body mass of 83.6 kg. As the THUMS model does not scale or morph based on individual biometric parameters, the kinematic data set chosen was the one with the closest fit to the THUMS average adult male with a height of 175 cm and body mass of 77 kg. To keep the validity of individual models, no additional scaling of the models was performed.

#### 2.1.2. Static Optimization Analysis

OpenSim online documentation (Available at: https://opensimconfluence.atlassian.net/wiki/spaces/OpenSim/overview (accessed 23 October 2025)) was used to plan the analysis process. The inverse kinematics method was used to apply the measured marker trajectories to the model. The file with analysis settings and relative marker weights was supplied with the model.

The STS transfer kinematics were then analyzed using the inverse dynamics analysis, which calculated joint torques during the observed motion. The ground reaction forces were applied to the left and right calcaneus. Kinematics data from inverse kinematics and ground reaction forces data were filtered with a low-pass filter of 6 Hz to suppress frequencies that did not originate from the human movement. The static optimization (SO) then solved for the minimal required muscle activation squared to produce muscle forces that result in previously determined joint torques.

#### 2.1.3. Residual and Reserve Actuators

In the Full Body Model 2016, the connection between the pelvis’ center of mass (COM) and the ground was defined as the first free joint with 6 DOF, 3 translational, and 3 rotational. Residual actuators were applied to the joint to bridge the discrepancies between the measured external forces and the accelerations calculated via the marker trajectories. Force actuators (Fx, Fy, Fz) and moment actuators (Mx, My, Mz) were applied to the translational and rotational DOF. The residual actuators were oriented according to the global coordinate system, as pictured in Figure 1. Three additional reserve moment actuators were applied to the spine.

As per OpenSim documentation, the loads in residual and reserve actuators should be as small as possible. Large reserve actuator loads imply that the model can’t reproduce the joint coordinates and torques using only the muscles included, but requires substantial external assistance. The loads these actuators produced at an activation value of 1.0 (α = 1.0) were set to 2 N and 2 Nm for force and moment actuators, respectively. These additional actuators can increase the activation value above 1. However, the analysis was not inclined to use that option unless necessary.

#### 2.1.4. Muscle and Joint Reaction Forces

The SO results were muscle activation and muscle force values during the STS transfer. With this, OpenSim calculated joint reaction forces in both hips and the lumbosacral joint in vector form. The reference coordinate system was the local pelvis coordinate system in the musculoskeletal model.

The muscle force orientation, however, had to be determined with the MuscleForceDirection plugin [29]. This plugin enables the extraction of direction vector components for all muscle forces attached to a selected body within the musculoskeletal model, with muscle attachments as points of origin. The local pelvis coordinate system was again considered as the reference coordinate system. The direction vectors’ components were then multiplied by muscle force magnitudes to determine the muscle force vector components. Finally, the data on the time dependency of muscle and joint reaction forces was prepared for use in LS-DYNA R12.

### 2.2. FEM Analysis

#### 2.2.1. FE Pelvis Model

**Figure 2:**
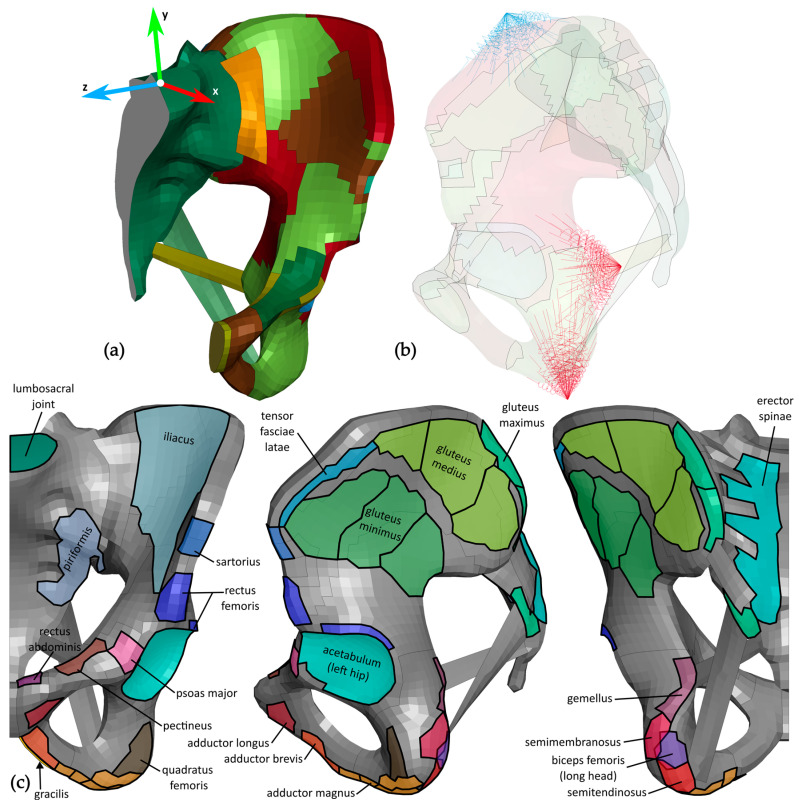
The FE model of the pelvis; (**a**) the reference coordinate system is pictured on the model, where parts with different material characteristics can be distinguished by color; (**b**) the model was made transparent to show support springs in the lateral view. The springs on the iliac crest are pictured in blue, and the springs on the posterior and inferior ischium in red; (**c**) the areas of muscle and joint forces are shown in the anterior, medial, and posterior views from left to right. The model is presented in grey, and muscle and joint forces are shown in color. The muscle attachments for muscles in the Full Body Model 2016, which were accounted for with multiple actuators (gluteus maximus, gluteus medius, gluteus minimus, and adductor magnus), are divided into sections of the same color. Each individual actuator force was assigned to one section.

From the selected THUMS model, the following parts, presented in Figure 2, were extracted into a keyword file by using LS-PrePost v4.7: sacrum, both hemipelvis bones, pubic symphysis, and the sacroiliac, sacrotuberous, and sacrospinous ligaments. A symmetry boundary condition was used to simplify the model, with the median plane adopted as the plane of symmetry and the left side of the pelvis retained.

Shell elements of varying thickness (0.5–3 mm) represented the cortical bone, while tetrahedral elements represented the trabecular bone. The pubic, sacrotuberous, sacrospinous, anterior sacroiliac, and posterior sacroiliac ligaments were represented by shell elements 2 mm thick. The pubic symphysis was modeled with hexahedral and interosseous sacroiliac ligaments with line elements. The size of the elements was approximately 4 mm and generally uniform. The model’s left side included 58 line, 3215 shell, and 16,490 solid elements. Various material models and parameters were used for different tissues (bones, ligaments, etc.). A general overview of material models, their parameters, and finite element formulations used is presented in Table 2. Detailed information about the model can be found on the THUMS repository (Available at: https://www.toyota.co.jp/thums/ (accessed 23 October 2025)).

#### 2.2.2. Coordinate Systems

The force vectors, obtained with SO, were defined in the local coordinate system of the pelvis in the musculoskeletal model. In LS-PrePost, a new coordinate system for the THUMS model was created and aligned with the one in the OpenSim model. The origin of the coordinate system was 2.5 mm above the base of the sacrum. The *x*- and *y*-axis lay in the sagittal plane, with the *x*-axis oriented anteriorly at a 40° angle above the base of the sacrum. The *z*-axis was oriented towards the right side of the body. The coordinate system is pictured in Figure 2.

#### 2.2.3. Boundary Conditions

Fixed supports can significantly influence structural analysis results through local stress and strain concentrations [30]. The symmetry plane and the following boundary conditions were utilized to avoid using them.

During the STS transfer, the pelvis accelerates and decelerates as its position changes. To reduce the displacement and rotation of the pelvis in the FEM analysis due to the lack of force equilibrium, the resultant force of all the muscle and joint reaction forces during the STS transfer was calculated. The force of the same magnitude but of an opposite direction as the resultant force was then applied across the entire cortical bone surface of the pelvis.

Due to the numerical approximation, a force imbalance persisted. To minimize the resulting movement, the pelvis was, based on the example of Ricci et al. [27], connected to the ground with three sets of 30 elastic springs with a stiffness of 7 N/mm—one on the iliac crest and the others on the posterior and inferior ischium. The spring stiffness was determined through an iterative process. It was kept large enough to prevent substantial rotation of the model and small enough to avoid accumulation of local stress concentrations. A small rotation (±3°) around the *z*-axis during the FEM analysis could not be avoided, but the model was checked for sensitivity to muscle force direction rotation around the *z*-axis, and it was confirmed that the results were not significantly affected by the pelvis rotation.

The muscle and joint reaction forces determined in OpenSim were applied to the cortical bone as loads, distributed across the nodes. The muscle loads were placed on the pelvis according to the muscle attachment sites as described by Neumann et al. [31]. These muscle attachment sites used for the FE model differed slightly from the ones in the musculoskeletal model and are presented in Figure 2. The magnitude of the force in the lumbosacral joint was halved to satisfy the symmetric boundary condition. To distribute the loads uniformly across the nodes, each load magnitude was scaled by 1/n, where n was the number of nodes to which the load was applied.

In total, 21 muscles represented by 30 OpenSim actuator forces were included in the study. Together with two joint reaction forces and the opposing resultant force, 33 forces were applied to the FE model, presented in Figure 2. The LS-DYNA R12 MPP single explicit solver was used for the FE analysis.

## 3. Results

The results will be presented in two parts. The first part will present muscle and joint reaction forces obtained through static optimization in OpenSim. The second part will focus on the stresses and strains in the pelvis during the STS transfer, determined through the FEM analysis in LS-DYNA.

The data is presented through the progress of the STS transfer process, as defined by Caruthers et al. [28] by modifying the phases used by Schenkman et al. [32]. The motion was divided into three phases: leaning forward (Phase 1), momentum transfer or liftoff (Phase 2), and extension (Phase 3).

### 3.1. Motion Analysis Results

The loads presented in the following chapter are shown in absolute values and values relative to the participant’s body weight (BW). Due to the forces on the left side of the pelvis being larger, all the muscles presented in this chapter are located on the left side of the body.

#### 3.1.1. Joint Reaction Forces

**Figure 3:**
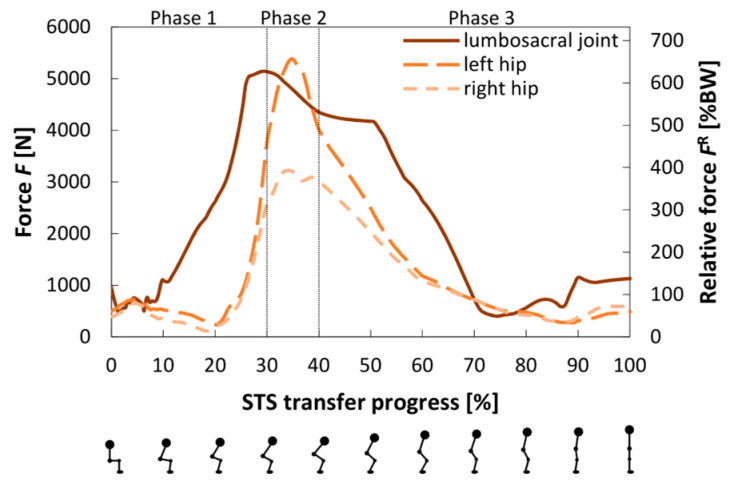
Reaction forces in hip joints and the lumbosacral joint during the STS transfer. Figures below the horizontal axis show the body position, which correlates with the state of the STS transfer progress.

The reaction forces in both hip joints and the lumbosacral joint during the STS transfer are presented in Figure 3. The magnitudes were small in the beginning stages when the participant was not moving. The first force to increase was the force in the lumbosacral joint when the participant began to lean forward, which required additional stabilization of the spine. The hip forces increased at the beginning of the chair liftoff. During the extension phase, the forces in all joints slowly decreased. As shown in Figure 3, the force in the left hip was larger than in the right hip. This was consistent with the ground reaction forces, which indicated that the participant transferred most of the weight during the motion to the left leg. The difference in the hip joint forces was then amplified through muscle joint compression.

#### 3.1.2. Loads in Reserve and Residual Actuators

**Figure 4:**
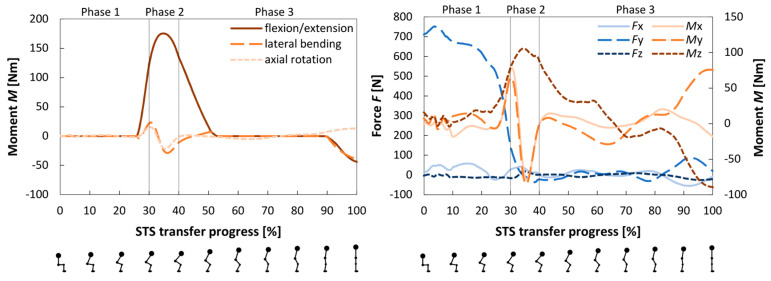
Activity of reserve (**left**) and residual (**right**) actuators during the STS transfer. Positive values for reserve actuators indicate spine extension, right-hand lateral bending, and left-hand axial rotation. The referenced coordinate system for the residual actuators is the fixed coordinate system in the Full Body Model 2016, pictured in Figure 2. Positive force values indicate action in positive directions of the coordinate system, and positive moments follow the right-hand rotation rule. Figures below the horizontal axis show the body position, which correlates with the state of the STS transfer progress.

Figure 4 shows the moments produced by the reserve actuators at the spine and the forces and moments generated by residual actuators between the pelvis COM and the ground. The reserve actuators in the hip, knee, and ankle joints remained below 0.5 Nm for the entire STS transfer, and the reserve actuators in the spine only activated during the lift-off phase. The residual force actuators around the pelvis remained below 100 N for most of the motion, except for the actuator working in the *y*-axis direction, which started above 700 N and dropped below 50 N during the lift-off phase. The residual moment actuators around the *x* and *y*-axis produced large oscillations during the lift-off phase. The residual actuator around the *z*-axis also reached its peak in the same phase to assist with the pelvic tilt.

#### 3.1.3. Muscle Forces

**Figure 5:**
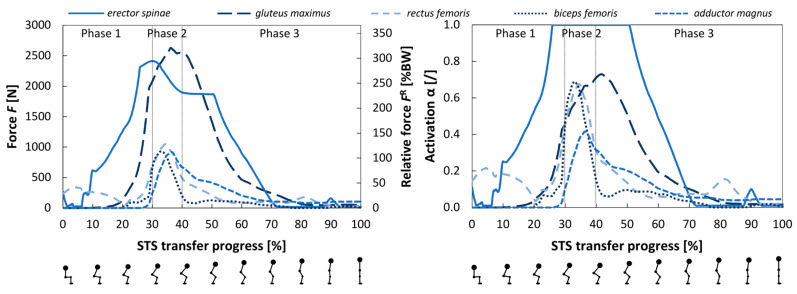
The forces (**left**) and activation (**right**) of the gluteus maximus, gluteus medius, erector spinae, rectus femoris, biceps femoris (long head), and adductor magnus muscles during the STS transfer. Figures below the horizontal axis show the body position, which correlates with the state of the STS transfer progress.

Presented in Figure 5 are forces and activation of the following muscles attached to the left side of the pelvis during the STS transfer: gluteus maximus, gluteus medius, erector spinae, rectus femoris, biceps femoris (long head), and adductor magnus. The forces of muscles with multiple actuators are presented as the sum of forces from all corresponding actuators, with differences in orientation disregarded. The activation of these muscles is presented as the average activation of all corresponding actuators. The data for other muscles can be found in the Supplementary Materials.

The muscle forces were consistent with the joint reaction forces in Figure 4. The force in the erector spinae was the first to increase. It was followed by muscles such as the gluteus maximus and adductor magnus, which assisted with hip extension. The activation of the presented muscles was relatively large (passing 0.6), with erector spinae even reaching full activation.

### 3.2. FE Analysis Results

Since the forces were larger on the left side of the body, that side was retained when applying symmetrical boundary conditions in the FEM analysis. The figures in this chapter, therefore, illustrate the left side of the pelvis.

**Figure 6:**
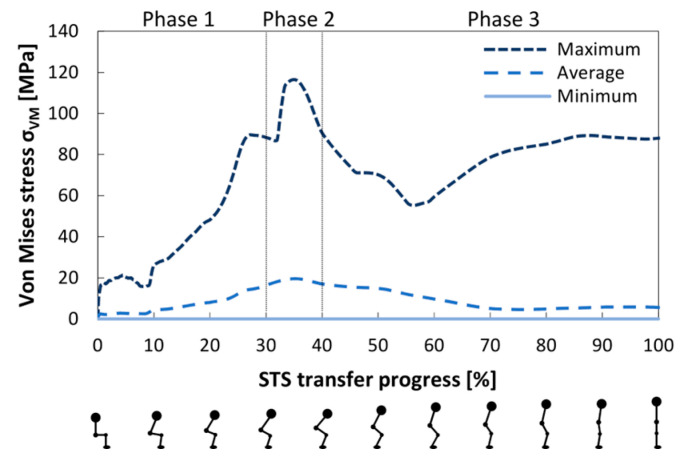
The peak, average, and minimum Von Mises stress in the pelvis during the STS transfer. Figures below the horizontal axis show the body position, which correlates with the state of the STS transfer progress.

**Figure 7:**
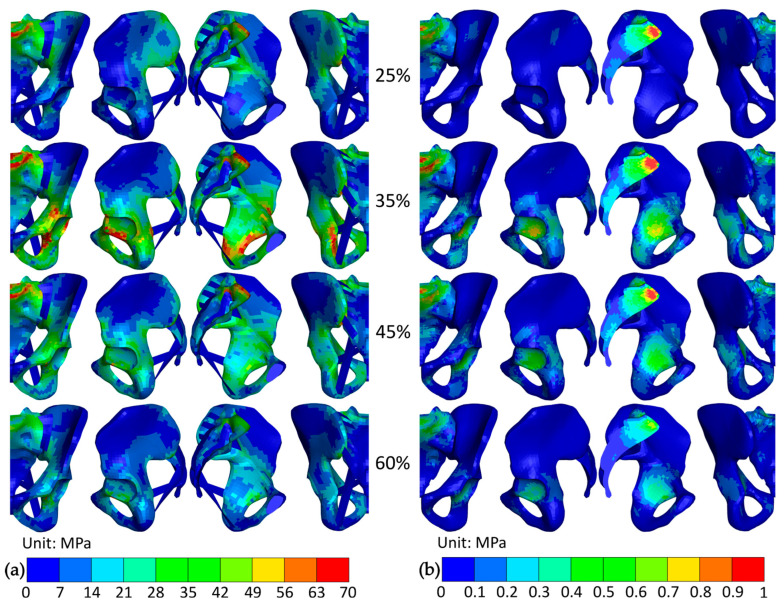
The Von Mises stress distribution in the (**a**) cortical and (**b**) trabecular bone at 25, 35, 45, and 60% of the STS transfer. The views from left to right are anterior, lateral, medial, and posterior.

The peak, average, and minimum Von Mises stresses within the pelvis during the STS transfer are presented in Figure 6. A detailed view of the state of the pelvis at 25, 35, 45, and 60% of the STS transfer is shown in Figure 7.

The largest Von Mises stress appeared during the liftoff phase, at 35% of the STS transfer progress, and remained in the cortical bone throughout the motion. As presented in Figure 7, the stresses above 28 MPa were located on the main load-bearing path in the pelvis. It consists of the shortest path between the lumbosacral and hip joint (ranging from the base of the sacrum through the sacroiliac joint, the arcuate line, the pecten pubis, and terminating at the acetabulum), through which the load from the torso is transmitted to the lower extremities, and the pubis, which completes the pelvic ring. They also appeared at the attachments of the gluteus maximus on the posterior inferior iliac spine and in the lesser sciatic notch on the ischium. The largest recorded Von Mises stress reached 118.7 MPa and was located between the posterior edge of the obturator foramen and the acetabulum.

In the trabecular bone, the largest Von Mises stress value reached 1.45 MPa and was located 4 mm beneath the central base of the sacrum. It coincided with the peak stress in the lumbosacral joint at 30% of the STS transfer. Stresses above 0.8 MPa appeared in the superior sacrum and beneath the acetabulum.

**Figure 8:**
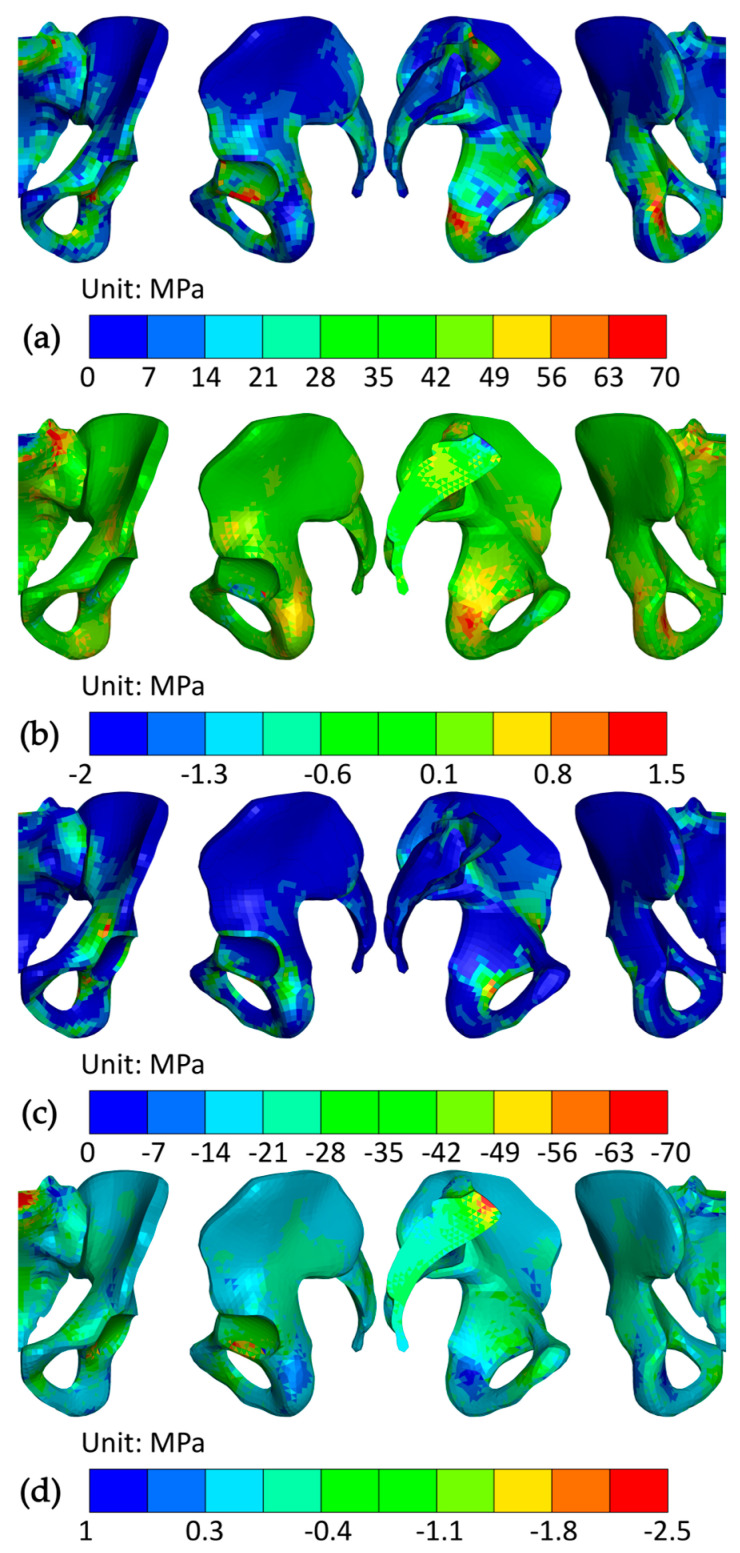
Stress in the pelvis at 35% of the STS transfer; (**a**) first principal stress in the cortical bone; (**b**) first principal stress in the trabecular bone; (**c**) third principal stress in the cortical bone; and (**d**) third principal stress in the trabecular bone. The views from left to right are anterior, lateral, medial, and posterior.

To identify the prevalent compressional and tensional stresses, the first and third principal stresses in both cortical and trabecular bone at 35% of the STS transfer are presented in Figure 8. In the cortical bone, the largest compressional loads appeared on the posterior and superior edges of the obturator foramen and on the iliopubic eminence. The largest tensile loads were present in the acetabular notch, at the same location as peak Von Mises stress, reaching 86.9 MPa. Increased tensional stresses were also generated in the lesser sciatic notch and on the sacrum (beneath the anterior edge of the base and on the anterior ala). Generally, cortical bone was loaded more in tension than compression, opposite to the trabecular bone. There, the largest compressional loads appeared directly beneath the cortical bone in the base of the sacrum and reached 3.1 MPa, while the largest tensile loads were located at the sacral ala.

**Figure 9:**
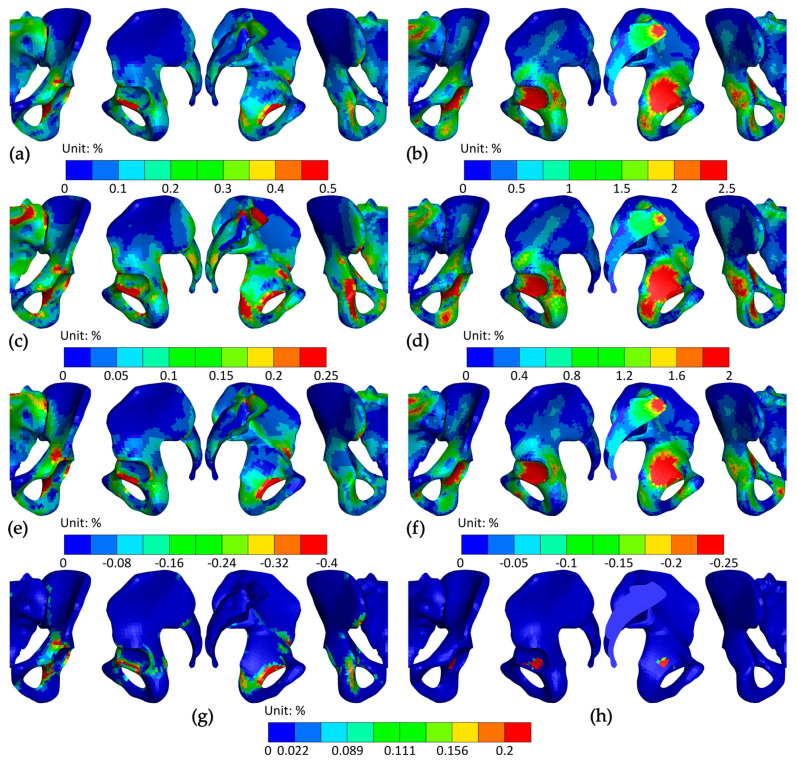
Strains in the pelvis at 35% of the STS transfer; (**a**) mean integration point effective strain in cortical bone; (**b**) mean integration point effective strain in trabecular bone; (**c**) peak principal strain in cortical bone; (**d**) peak principal strain in trabecular bone; (**e**) minimum principal strain in cortical bone; (**f**) minimum principal strain in trabecular bone; (**g**) plastic strain in cortical bone; and (**h**) plastic strain in trabecular bone. The views from left to right are anterior, lateral, medial, and posterior.

Strains in the cortical bone, presented in Figure 9, were small, except for the transition from the acetabulum to the obturator foramen, where they reached 2.0%. The strains in the acetabulum were mostly in tension, and in the obturator foramen, mostly in compression. The most strained parts of the trabecular bone were in the base of the sacrum and the body of the ilium beneath the acetabulum. The peak elastic effective strain value of 7% was generated in the latter. The third principal strain outweighed the first principal strain in the above locations, which indicates that trabecular bone was loaded predominantly in compression.

## 4. Discussion

The pelvis was most heavily loaded at 35% of the STS transfer (Figure 6). The discussion will therefore focus on the state of the pelvis at that stage of the motion. Joint and muscle loads determined in OpenSim will be discussed in the first part, followed by a discussion on the FE analysis.

As primary boundary conditions for the FEM analysis, muscle and joint reaction forces strongly influenced the determined stresses and strains. Caruthers et al. [28] took several steps to ensure the accuracy of their model. They described the procedure they used to minimize the influence of residual and reserve actuators, which resulted in reserve actuators remaining within the limits recommended in OpenSim documentation, so below 75 Nm, for the entirety of the analyzed STS transfer. The only exception was the spine flexion/extension actuator during the liftoff phase. Together with high activation of the erector spinae muscle, this indicates the Caruthers’ model could benefit from a review of the back musculature and/or joints modelling. Residual force actuators’ main task was to substitute the reaction forces under the chair, which were not measured by Caruthers et al., but outside of the actuator’s *F*_y_ large initial magnitude, remained within the previously mentioned acceptable limits.

Caruthers et al. also reported matching the muscle activations in the model with the recorded EMG data. Their report, however, did not include data on joint reaction forces, which are the main discrepancies between our simulation data and experimental results of other studies. It appears that the peak joint forces predicted by Caruthers’ model during an STS transfer exceed expected values obtained from studies measuring in vivo joint forces with instrumented implants and are closer to peak loads during jogging, but still physiologically acceptable [9,10].

The increase in joint reaction forces likely resulted from the large estimated muscle forces. Establishing reliable muscle force values is a challenge in its own, since the literature on actual in vivo muscle forces is sparse. This can be observed from the fact that FEM studies of the pelvis during gait have used the same set of data for almost three decades [33,34,35], while the methodology for deriving this data is even older [36]. This still makes the Caruthers’ model one of the newest data sets available for studies like the one presented here, but it can be assumed that the joint and muscle loads determined by the Caruthers’ model are the primary reasons for increased stresses in our FEM analysis compared to those estimated by most other similar studies [27,30,33,34,35,37,38]. The hip and/or lumbar forces used in those studies were 10–40% of those predicted by the Caruthers’ model, and the stresses and strains predicted by our FE analysis are consistent with that increase.

The stress–strain distribution patterns in the pelvis during the STS transfer determined by our analysis are more consistent with the existing literature. The Von Mises stress distribution is interestingly similar to those reported by gait and standing analyses by Dalstra and Huiskes [33], Phillips et al. [30], Watson et al. [25], Ricci et al. [27] and Volinski et al. [34]. It was expected that the stress increase would occur on the main load-bearing path in the cortical bone. The analysis also showed the stress increased at several other locations, which coincided with the findings of previous studies. Increased stress in the acetabular notch and on the superior edge of the obturator foramen was noticed by Anderson et al. [37], Watson et al. [25], and Volinski et al. [34], at the attachments of the gluteus maximus on the ilium by Dalstra and Huiskes [33], Watson et al. [25] and Volinski et al. [34], and in the lesser sciatic notch by Volinski et al. [34].

Our FE analysis showed that during the STS transfer, the pelvis was compressed in the lateral direction, thus stretching in the anterior–posterior direction. The greater sciatic notch widened, and the base of the sacrum moved anteriorly. The forces in the hip joint and from the adductor magnus and biceps femoris forced the inferior pubis and ischium to bend in the anterior direction, which caused large Von Mises stresses in the lesser sciatic notch, in the acetabular notch, and on the edges of the obturator foramen.

Our FE analysis confirmed observations made by Dalstra and Huiskes [33] and Phillips et al. [30], who showed that the locations of the largest stresses in trabecular bone do not coincide with those in cortical bone. At the base of the sacrum, the largest stresses in the cortical bone appeared on the edges, while the largest stress in the trabecular bone appeared beneath the central base. In the moment of the largest hip contact force (at 35% of the STS transfer), the largest stress in the trabecular bone appeared beneath the central acetabulum, and the largest stress in the cortical bone appeared at the edge of the acetabulum. The joint reaction forces significantly influenced trabecular bone stresses, as the largest stress at both locations coincided with the largest joint reaction force.

Some potential improvements to the study were identified. Firstly, differences in the geometry between the pelvis in the Full Body Model 2016 and THUMS were disregarded. Anatomically corrected muscle attachments were used, which also increased the differences between the SO and FEM analyses, but, on the other hand, brought the FE setup closer to physiological conditions.

The model loading could benefit from more realistic joint force magnitudes. Other studies have avoided calculating them directly, instead using measured data [30,33,34] or different supports [25,26,27,39] to supplement muscle forces obtained from separate sources. Hip joint modeling could be improved as well, by including the femoral head and applying the force to the joint through it, instead of directly to the nodes of the acetabulum, as several studies have shown the importance of attention to detail in the modeling of the hip joint [40,41,42].

Additionally, the geometric and material models in the THUMS pelvis used several simplifications that might not be suitable for an analysis focusing on a specific body part. Certain geometries, like the acetabulum and the sacrum, were modified for easier meshing and faster computation. The material models used were also limited in certain aspects. While modern studies employ material models with different behaviors in tension and compression [43,44], the THUMS model incorporates simpler viscoelastic models that do not depend on the direction of loading. For detailed modeling, the varying density of trabecular bone [45] should also be considered [43,44,46]. THUMS includes a homogenous trabecular bone with a uniformly assigned elastic modulus of 15 MPa, whereas some studies have considered values ranging from 50 to 500 MPa [47]. A stiffer trabecular bone would provide better support to the cortical bone, resulting in a different stress–strain distribution.

## 5. Conclusions

This study aimed to be the first to analyze the stress–strain state of the pelvis during the sit-to-stand transfer. Directions and magnitudes of muscle and joint forces during the motion were predicted in OpenSim with static optimization analysis. A unique set of boundary conditions was utilized to stabilize the FE model, enabling a dynamic structural analysis of the pelvis under physiological loads during the motion. The results provide important insights into the global behavior of the pelvic ring during sit-to-stand transfer, indicate the locations of concentrated stress, and could be used to assess the current pelvic fracture fixation procedures. This work could benefit from improvements in both determining muscle and joint forces during movement and in the geometry and material formulations. The case presented here is single-motion and subject-specific, and yet this study demonstrates a feasible process with vast potential for patient-specific predictive modeling using physiological loading conditions, which we intend to further develop for use in medical and engineering fields and beyond.

## Figures and Tables

**Table 1:** List of muscles attached to the pelvis included in the Full Body Model 2016 and the number of actuators they are represented by.

Muscle	Nr. of Actuators	Muscle	Nr. of Actuators
Adductor brevis	1	Pectineus	1
Adductor longus	1	Piriformis	1
Adductor magnus	4	Psoas major *	1
Biceps femoris (long head)	1	Quadratus femoris	1
Erector spinae	1	Rectus abdominis	1
Gemellus	1	Rectus femoris	1
Gluteus maximus	3	Sartorius	1
Gluteus medius	3	Semimembranosus	1
Gluteus minimus	3	Semitendinosus	1
Gracilis	1	Tensor fasciae latae	1
Iliacus	1		

* Psoas major does not truly attach to the pelvis, but it does travel over the superior pubic ramus and therefore exerts a force on the pelvis.

**Table 2:** List of anatomical structures in the FE model with Young’s modulus, Poisson number, and a general description of the assigned material model. Detailed information about the model can be found on the THUMS repository (Available at: https://www.toyota.co.jp/thums/ (accessed 23 October 2025)).

Material		E [MPa]	ν [/]	Material Model	LS-DYNA Material Code	LS-DYNA Element Formulation
Trabecular bone	Sacrum	40	0.45	elasto-plastic	MAT_105	Solid 13 (tet *)
	Hemipelvis	15	0.45	elasto-plastic	MAT_105	Solid 13 (tet *)
Cortical bone	Sacrum	13,020	0.3	elasto-plastic	MAT_081	Shell 16
	Hemipelvis	17,300	0.3	elasto-plastic	MAT_024	Shell 16
Pubic symphysis		0.05	/	nonlinear elastic	MAT_057	Solid 1 (hex **)
Pubic ligaments		20	0.4	hyperelastic	MAT_024	Shell 16
Sacrotuberous ligament		20	0.4	hyperelastic	MAT_024	Shell 16
Sacrospinous ligament		20	0.4	hyperelastic	MAT_024	Shell 16
Anterior and posterior SI ligaments		20	0.4	hyperelastic	MAT_024	Shell 16
Interosseous SI ligaments		5000	0.3	rigid	MAT_020	Beam 3

* tet—tetrahedral elements, ** hex—hexahedral elements.

## Data Availability

The raw data supporting the conclusions of this article will be made available by the authors on request.

## Supplementary Materials

The following supporting information can be downloaded at: https://www.mdpi.com/article/10.3390/bioengineering12121328/s1, Figure S1: The forces and activation of the psoas, semimembranosus, gluetus medius, iliacus and quadratis femoris muscles during the STS transfer; Figure S2: The forces and activation of the psoas, semimembranosus, gluetus medius, iliacus and quadratis femoris muscles during the STS transfer; Figure S3: The forces and activation of the gluteus minimus, pectineus, semitendinosus, tensor fasciae latae and gracilis muscles during the STS transfer; Figure S4: The force and activation of the rectus abdominis muscle during the STS transfer; Table S1: Overview of muscle activity during the STS transfer.

## Abbreviations

The following abbreviations are used in this manuscript:

EMG	Electromyography
MC	Muscle Contraction
FEM	Finite Element Method
FE	Finite Element
STS	Sit-to-Stand
THUMS	Total HUman Model for Safety
DOF	Degrees of Freedom
SO	Static Optimization
COM	Center of Mass
BW	Body Weight
