# Supplementary material for: The Stress–Strain State in the Pelvis During Sit-to-Stand Transfer
Source: Bioengineering (Basel). 2025 Dec 5;12(12):1328. doi: 10.3390/bioengineering12121328 (PMC12729763; doi:10.3390/bioengineering12121328)
Supplement: Supplementary file 1 [file bioengineering-12-01328-s001.zip › bioengineering-3976511-supplementary.pdf]

Supplementary Material

The Stress–Strain State in the Pelvis During Sit-to-Stand Transfer

Urban Žnidaršič [1], Andrej Žerovnik [1], Matevž Tomaževič [2] and Robert Kunc [1,*]

[1] Chair of Modeling in Engineering Sciences and Medicine, Faculty of Mechanical Engineering, University of Ljubljana, Kongresni trg 12, 1000 Ljubljana, Slovenia
[2] Division of Surgery, Department of Traumatology, University Medical Centre Ljubljana, Zaloška cesta 7, 1000 Ljubljana, Slovenia
* Correspondence: robert.kunc@fs.uni-lj.si

1. Muscle force and activation profiles during the STS transfer

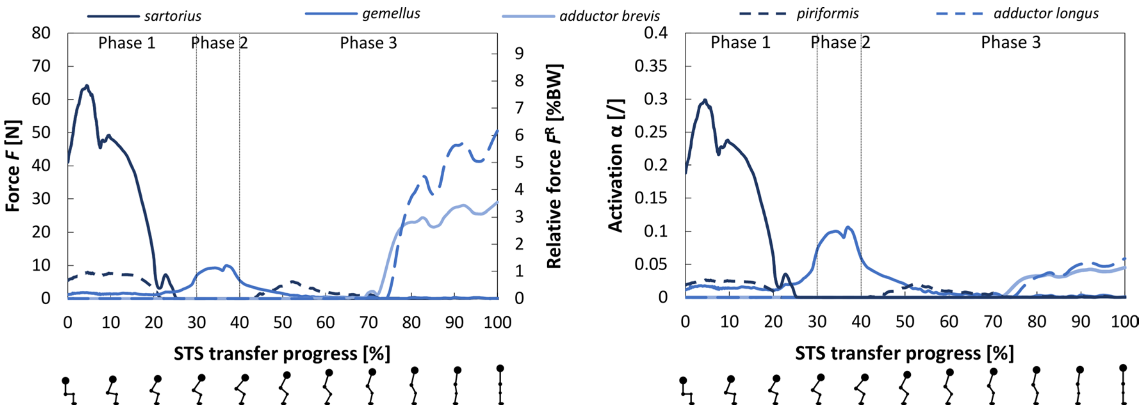

Figure S1. The forces (**left**) and activation (**right**) of the psoas, semimembranosus, gluetus medius, iliacus and quadratis femoris muscles during the STS transfer. Figures below the horizontal axis show the position of the body which corresponds with the state of the STS transfer progress.

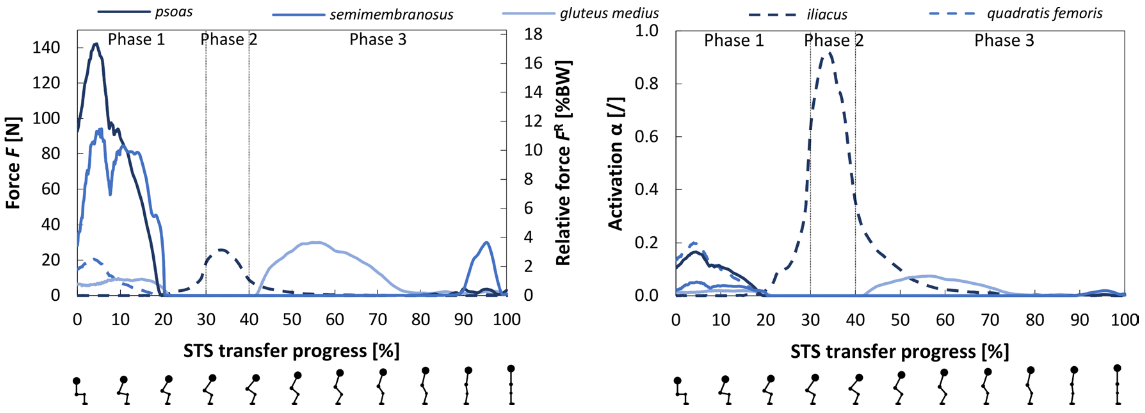

Figure S2. The forces (**left**) and activation (**right**) of the psoas, semimembranosus, gluetus medius, iliacus and quadratis femoris muscles during the STS transfer. Figures below the horizontal axis show the position of the body which corresponds with the state of the STS transfer progress.

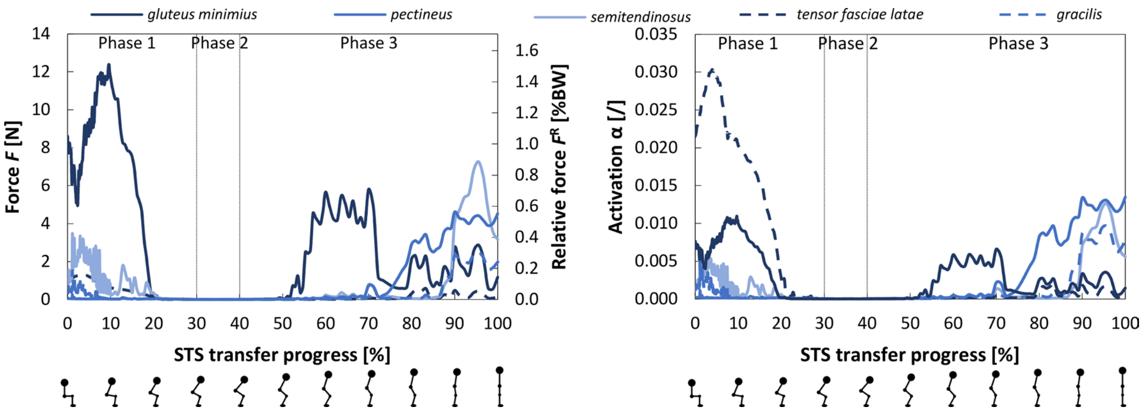

Figure S3. The forces (**left**) and activation (**right**) of the gluteus minimus, pectineus, semitendinosus, tensor fasciae latae and gracilis muscles during the STS transfer. Figures below the horizontal axis show the position of the body which corresponds with the state of the STS transfer progress.

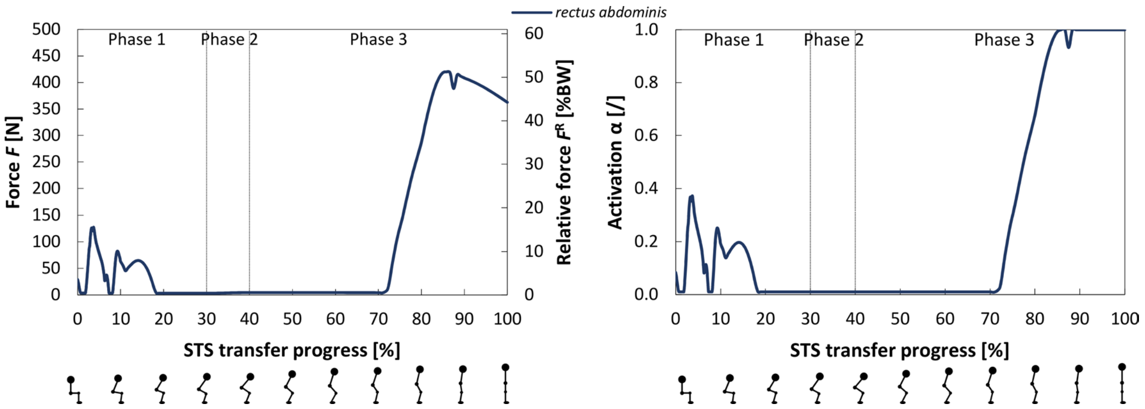

Figure S4. The force (**left**) and activation (**right**) of the rectus abdominis muscle during the STS transfer. Figures below the horizontal axis show the position of the body which corresponds with the state of the STS transfer progress.

Table S1. Overview of muscle activity during the STS transfer.

**Muscle**	**Total time of activation above 0.02 [s]**	**Highest activation level [/]**	**Average force magnitude during activation [N]/[% BW]**	**Maximal force magnitude [N]/[% BW]**	**Moment of maximal force occurance during STS transfer [%]**
Adductor brevis	0.19	0.05	24 / 2.9	29 / 3.5	100
Adductor longus	0.17	0.06	39 / 4.8	51 / 6.2	100
Adductor magnus	0.68	0.42	421 / 51.3	919 / 112.1	37
Biceps femoris (long head)	0.63	0.69	251 / 30.6	931 / 113.5	33
Erector spinae	1.15	1.00	1107 / 135.0	2414 / 294.3	30
Gemellus (inferior & superior)	0.36	0.11	6 / 0.7	10 / 1.2	37
Gluteus maximus	0.77	0.73	1144 / 139.5	2628 / 320.4	36
Gluteus medius	0.27	0.07	187 / 22.8	246 / 30.0	55
Gluteus minimus	0	0.01	/	/	/
Gracilis	0	0.01	/	/	/
Iliacus	0.69	0.20	115 / 14.0	169 / 20.6	4
Pectineus	0	0.01	/	/	/
Piriformis	0.66	0.03	7 / 0.9	8 / 1.0	5
Psoas major	0.69	0.17	108 / 13.2	142 / 17.3	4
Quadratus femoris	0.58	0.92	65 / 7.9	211 / 25.7	33
Rectus abdominis	0.76	1.00	131 / 16.0	420 / 51.2	86
Rectus femoris	1.53	0.67	291 / 35.5	1048 / 127.8	34
Sartorius	0.77	0.30	48 / 5.8	64 / 7.8	4
Semimembranosus	0.68	0.05	73 / 8.9	94 / 11.5	5
Semitendinosus	0	0.01	/	/	/
Tensor fasciae latae	0.60	0.03	1 / 0.1	1 / 0.1	3
